# Duplicate Abalone Egg Coat Proteins Bind Sperm Lysin Similarly, but Evolve Oppositely, Consistent with Molecular Mimicry at Fertilization

**DOI:** 10.1371/journal.pgen.1003287

**Published:** 2013-02-07

**Authors:** Jan E. Aagaard, Stevan A. Springer, Scott D. Soelberg, Willie J. Swanson

**Affiliations:** 1 Department of Genome Sciences, University of Washington, Seattle, Washington, United States of America; 2 Department of Cellular and Molecular Medicine, University of California San Diego, La Jolla, California, United States of America; University of Wisconsin - Madison, United States of America

## Abstract

Sperm and egg proteins constitute a remarkable paradigm in evolutionary biology: despite their fundamental role in mediating fertilization (suggesting stasis), some of these molecules are among the most rapidly evolving ones known, and their divergence can lead to reproductive isolation. Because of strong selection to maintain function among interbreeding individuals, interacting fertilization proteins should also exhibit a strong signal of correlated divergence among closely related species. We use evidence of such molecular co-evolution to target biochemical studies of fertilization in North Pacific abalone (*Haliotis* spp.), a model system of reproductive protein evolution. We test the evolutionary rates (*d*_N_/*d*_S_) of abalone sperm lysin and two duplicated egg coat proteins (VERL and VEZP14), and find a signal of co-evolution specific to ZP-N, a putative sperm binding motif previously identified by homology modeling. Positively selected residues in VERL and VEZP14 occur on the same face of the structural model, suggesting a common mode of interaction with sperm lysin. We test this computational prediction biochemically, confirming that the ZP-N motif is sufficient to bind lysin and that the affinities of VERL and VEZP14 are comparable. However, we also find that on phylogenetic lineages where lysin and VERL evolve rapidly, VEZP14 evolves slowly, and vice versa. We describe a model of sexual conflict that can recreate this pattern of anti-correlated evolution by assuming that VEZP14 acts as a VERL mimic, reducing the intensity of sexual conflict and slowing the co-evolution of lysin and VERL.

## Author Summary

Interacting sperm and egg proteins must co-evolve to maintain compatibility at fertilization, so their divergence among species should be correlated—lineages with rapidly evolving sperm proteins should have rapidly evolving egg proteins. We use this expectation to target biochemical studies of fertilization in a model system (abalone). We study a discrete functional domain (ZP-N) found in a pair of duplicated egg coat proteins, and we find the ZP-N motif from both proteins bind sperm lysin (a protein important for sperm passage of the egg coat) in a similar fashion. ZP-N is a feature of vertebrate and invertebrate egg coat proteins, as well as yeast mating recognition proteins, demonstrating its broad significance in sexual reproduction. Unexpectedly, we find that the ZP-N motifs of VEZP14 and VERL exhibit inverse patterns of co-evolution with lysin, suggesting that these duplicates may have opposite functions in fertilization. Using computer simulations, we model a novel explanation for this pattern whereby VEZP14 acts as a decoy of VERL in order to decrease the effective amount of sperm lysin and slow the rate of fertilization. Such molecular mimicry could complement other well-established fertilization blocks that females use to control rates of fertilization and limit polyspermy.

## Introduction

Reproductive genes are often identified in genome-wide scans as targets of positive selection, and some are among the most rapidly evolving proteins known [1]–[4]. Their rapid adaptive evolution is observed in a broad range of organisms [5], [6], which is remarkable given the central importance of reproductive compatibility to organismal fitness. Moreover, experiments show that even a few amino-acid substitutions among cognate fertilization proteins can cause reproductive isolation [7], and so rapid divergence takes place in the context of strong selection to maintain functional interaction. Because cognate sperm and egg proteins must co-evolve to maintain compatibility, their divergence should result in correlated evolutionary rates – on lineages where females evolve rapidly, males should also evolve rapidly. This expectation of correlated evolution between males and females underlies a two-pronged approach to investigating molecular interactions at fertilization: we can use evolutionary signals of co-evolution to focus genetic and biochemical assays on molecules that are particularly likely to functionally interact.

Correlation in the ratio of non-synonymous to synonymous nucleotide substitution (*d*_N_/*d*_S_, or ω) has been shown to reflect known protein-protein interactions [8] including those between reproductive proteins of the free-spawning marine gastropod abalone (*Haliotis* spp. [9]). Abalone are a prominent model system for the study of reproductive proteins, and one of the few cases in which cognate sperm and egg fertilization proteins have been identified [10]–[12]. Abalone eggs are surrounded by a raised Vitelline Envelope (VE) comprised of tightly compacted fibers [13] that present a species-specific barrier to sperm entry [14]. Abalone sperm de-condense the VE fibers to create a hole in the VE via a non-enzymatic mechanism that involves binding between positively charged ∼16 kiloDalton (kDa) sperm lysin [13] and a large (>1000 kDa) VE glycoprotein (the Vitelline Envelope Receptor for Lysin, VERL) [10]. VERL contains an array of ∼22 negatively charged ∼150 amino acid tandem repeats, each of which is believed to contain a lysin binding domain [10], [15]. Stoichiometry

of VE dissolution indicates that two lysin molecules bind each repeat [10], in support of a model whereby lysin dimers out compete hydrophobic interactions among intermolecular VERL repeats and unravel VE fibers in a zipper-like fashion through surface structure and electrostatic interactions [16].

Both lysin and VERL show recurrent adaptive divergence among the 8 abalone species that diverged <18 million years ago in the North Pacific [17]. Positive selection on lysin residues corresponds to domains known to mediate species-specific VE dissolution [7], and was previously shown to be restricted to the two N-terminal repeats of VERL [18] consistent with observations that initiation of VE dissolution is the rate-limiting step which serves to reproductively isolate abalone species [16]. Consistent with both biochemical and evolutionary analyses implicating co-evolution between lysin and VERL, adaptive divergence of lysin and the N-terminal VERL repeats (as measured by *d*_N_/*d*_S_) has been shown to be positively correlated across branches of the abalone phylogeny [9].

Many of the constituent proteins of the abalone VE have been characterized and are known to also evolve under positive selection [19], [20], including a paralog of VERL called Vitelline Envelope Zona Pellucida 14 (VEZP14) [19]. VEZP14 is one of >30 abalone VE proteins that contain a polymerization module (the ZP domain) [21] common among both invertebrate and vertebrate egg coats. Uniquely, it also has a single N-terminal domain with homology to the VERL repeats and which has also been the target of positive selection [19]. Structural models [22] demonstrate that this N-terminal domain of VEZP14 and the VERL repeats all contain a motif corresponding to a novel β-sandwich fold of the immunoglobulin (Ig) superfamily of proteins named for the N-terminal portion of the ZP domain from which the structure was resolved (ZP-N) [23]. Remarkably, this fold is a feature of mouse egg coat sperm receptors ZP2 and ZP3 [23] as well as yeast mating proteins α-Agglutinin/Sag1p [22], [24], demonstrating its likely importance in gamete recognition and sexual reproduction across multi-cellular organisms.

Here, we use molecular co-evolutionary analyses in combination with biochemical assays to investigate the molecular interactions between abalone sperm and egg coat proteins during fertilization. A strong signal of co-evolution specifically between lysin and ZP-N motifs focus our biochemical assays that demonstrate the ZP-N motif is sufficient for binding of lysin. Our co-evolutionary analyses also reveal a surprising pattern (correlated evolution between lysin and VERL, but anti-correlated evolution with VEZP14) that suggests unexpected modes of interaction among these fertilization proteins not evident from binding assays. We develop a population model to explore one such interaction whereby VEZP14 functions as a VERL mimic, binding excess lysin and reducing the intensity of sexual conflict by reducing fertilization rate.

## Results/Discussion

Two distinct but complementary approaches identify ZP-N from duplicated abalone VE proteins, VERL and VEZP14, as sperm lysin receptor motifs. First, evolutionary rates as measured by the ratio of non-synonymous to synonymous nucleotide substitution (*d*_N_/*d*_S_) are correlated between lysin and ZP-N from both genes across the evolution of North Pacific abalone species – a pattern not seen even with other domains of these genes. Second, affinity assays utilizing surface plasmon resonance (SPR) quantification of association and dissociation rates between green abalone lysin and expressed ZP-N motifs shows binding kinetics are comparable for VERL and VEZP14. While our co-evolutionary analyses are shown to be a useful tool in focusing these more detailed biochemical studies confirming ZP-N is sufficient to bind lysin, they may also offer novel insight into how egg coat proteins mediate sperm passage of the VE during abalone fertilization. We first present results from our co-evolutionary and biochemical studies, after which we discuss implications for models of abalone fertilization.

### The evolutionary rates of abalone lysin and the ZP-N motifs of VERL and VEZP14 are correlated

**Figure 1:**
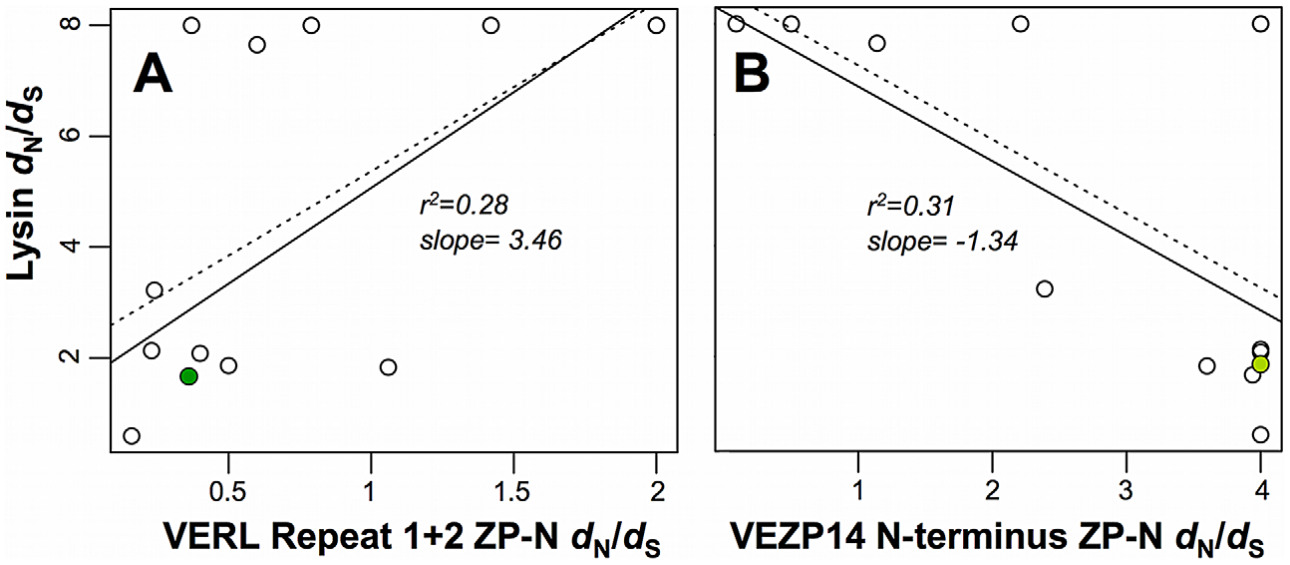
Evolutionary rates between sperm and egg coat proteins are correlated. The ratio of non-synonymous to synonymous nucleotide substitutions (*d*_N_/*d*_S_ or ω) for sperm acrosomal (lysin) or duplicated egg coat (VERL and VEZP14) proteins were estimated using the branch model of codeml [49] with gene trees following the topology of [9]. Values of ω for each branch of the lysin tree are plotted on the y-axis relative to x-axis values for the corresponding ZP-N motifs from (A) VERL repeats 1 and 2, or (B) VEZP14. Simple linear regression models either weighted (solid lines) or unweighted (dashed lines) by branch length as in [9] were fit to the data [50], and show a significant positive or negative correlation between lysin and VERL (p = 0.06, weighted; p = 0.02, unweighted) or VEZP14 (p = 0.05, weighted; p = 0.02 unweighted), respectively. Data points with green fill correspond to the branches leading to green abalone used in binding experiments (Figure 2).

Because cognate sperm and egg proteins must co-evolve to maintain reproductive compatibilities, their rapid divergence among species is predicted to result in correlated evolutionary rates along phylogenetic lineages. We expand upon an earlier study that demonstrated such a positive correlation between abalone lysin and the combined first three full (∼150 AA) repeats of VERL [9]. Here we focus on discrete ZP-N sequences recently proposed [22] to constitute a sperm protein binding motif, specifically ZP-N from VERL repeats 1 and 2 (numbered consecutively from VERL's N-terminus) as well as VEZP14. As previously, we tested for correlations by fitting linear regression models to point estimates of branch-specific *d*_N_/*d*_S_ ratios across the phylogeny of North Pacific abalone, a conservative but robust approach in the absence of genome-wide data necessary to implement more powerful models of correlated evolution [8], [9]. While discrete ZP-N motifs of the first and second VERL repeats analyzed individually do not show a statistically significant positive correlation in *d*_N_/*d*_S_ with lysin, consistent with a relatively weak signal of positive selection on each individually relative to ZP-N from VEZP14 (Table 1; and see below), analysis of the concatenated ZP-N motifs from VERL repeats 1 and 2 shows the predicted positive correlation as having modest statistical support based even on the more conservative weighted linear regression model (slope = 3.46, r^2^ = 0.28, p = 0.06 for; Figure 1A). Similarly, we find evidence of a correlation in *d*_N_/*d*_S_ between the ZP-N motif of VEZP14 and lysin – notably in contrast with lysin-VERL, a weighted linear regression identifies the lysin-VEZP14 correlation as negative (slope = −1.34, r^2^ = 0.31, p = 0.05). We discuss possible explanations for a positive -vs- negative correlation in *d*_N_/*d*_S_ with lysin in detail below in the context of models of abalone fertilization. To summarize: (*i*) positive selection is a feature of VERL repeats 1 and 2 as well as the ZP-N motif of VEZP14; (*ii*) the rate of evolution driven by positive selection is somewhat higher for the VEZP14 ZP-N motif; and (*iii*) whereas the rate of evolution along phylogenetic lineages is correlated between lysin and VERL (slow with slow, or fast with fast), lysin and VEZP14's evolutionary rates are anti-correlated (slow with fast).

**Table 1:** Evidence of positive selection among abalone sperm lysin and egg coat protein ZP-N motifs.

Gene	Sequence Length (codons)	Tree Length	2 Δ lnL (M8a –vs- M8)	p-value	% Sites Under Selection	*d*_N_/*d*_S_ (Model 8)
Lysin	136	1.9	88.7	<0.01	33%	7.5
VERLFull Repeat 1+2	260	0.8	7.2	0.01	19%	2.4
VERLRepeat 1+2 ZP-N	209	0.6	1.6	0.21	—	—
VERLRepeat 1 ZP-N	102	0.6	0.01	0.91	—	—
VERLRepeat 2 ZP-N	107	0.7	2.8	0.09	—	—
VEZP14N-terminal ZP-N	92	1.2	50.8	<0.01	19%	10.8

Nucleotide sequences of protein coding regions were analyzed using codeml in the PAML computer package [49]. Positive selection on lysin has been extensively studied previously (see, e.g. [46],), and results are provided here for ease of reference. Functional motifs of VERL were tested in several ways including: (*i*) analysis of entire repeats 1 and 2 of VERL as in [18]; (*ii*) by combined analyses of discrete ZP-N motifs [22] from repeat 1 and 2 of VERL; or (*iii*) analyzing individual ZP-N motifs from repeat 1 or 2. Tree lengths are calculated from the number of substitutions per codon. Likelihood-ratio tests of codon substitution models without -vs- with the possibility of positive selection (M8a or M8, respectively) support pervasive positive selection (ω>1) among lysin as well as the ZP-N motif of VEZP14, but evidence for VERL is weaker based on a significant test statistic only when analyzing full repeats previously shown to be under positive selection [18].

The correlated rates of evolution we find between lysin and the ZP-N motifs of VERL and VEZP14 are unique among the comparisons we analyzed. As seen previously for lysin and VERL [9], in our current work two control genes with no known function in reproduction also show no evidence of correlation in *d*_N_/*d*_S_ with the VEZP14 ZP-N motif (Figure S1A, S1B). In the absence of genome-wide data this provides only limited support for the significant correlations we identify. However, similar to previous studies of lysin and VERL [9] we also find the C-terminal ZP polymerization domain of VEZP14 exhibits a pattern of divergence comparable to control genes, with no evidence of a correlation in *d*_N_/*d*_S_ with either lysin or the N-terminal ZP-N motif itself (Figure S1C, S1D). This strongly supports the hypothesis of co-evolution specifically between ZP-N and lysin, implicating the motif as a discrete lysin binding sequence within VEZP14 as well as VERL repeats.

### Affinity of lysin for VERL and VEZP14 ZP-N motifs is comparable

The correlation in evolutionary rates specifically between lysin and ZP-N prompted us to explore the possibility of physical interactions using *in vitro* binding assays. We expressed discrete ZP-N motifs cloned from green abalone VERL and VEZP14 in a eukaryotic expression system to minimize the possibility of misfolding, followed by quantification of binding kinetics via SPR under realistic biological conditions reflective of the abalone fertilization environment (flowing seawater). While we were unsuccessful at obtaining protein from VERL repeats 2 and 3, ZP-N expression proteins of high purity from VERL repeat 1 and VEZP14 were obtained (Figure S2A, S2B) and validated based on multiple unique peptides via shotgun proteomic sequencing. Expression proteins showed evidence of differential addition of N-linked polysaccharides based on digestion with PNGaseF (Figure S2A, S2B) which may deviate from that seen among native VE components [25], but experimental evidence supports amino acid divergence as the primary determinant affecting specificity of lysin's dissolution of VEs [7].

**Figure 2:**
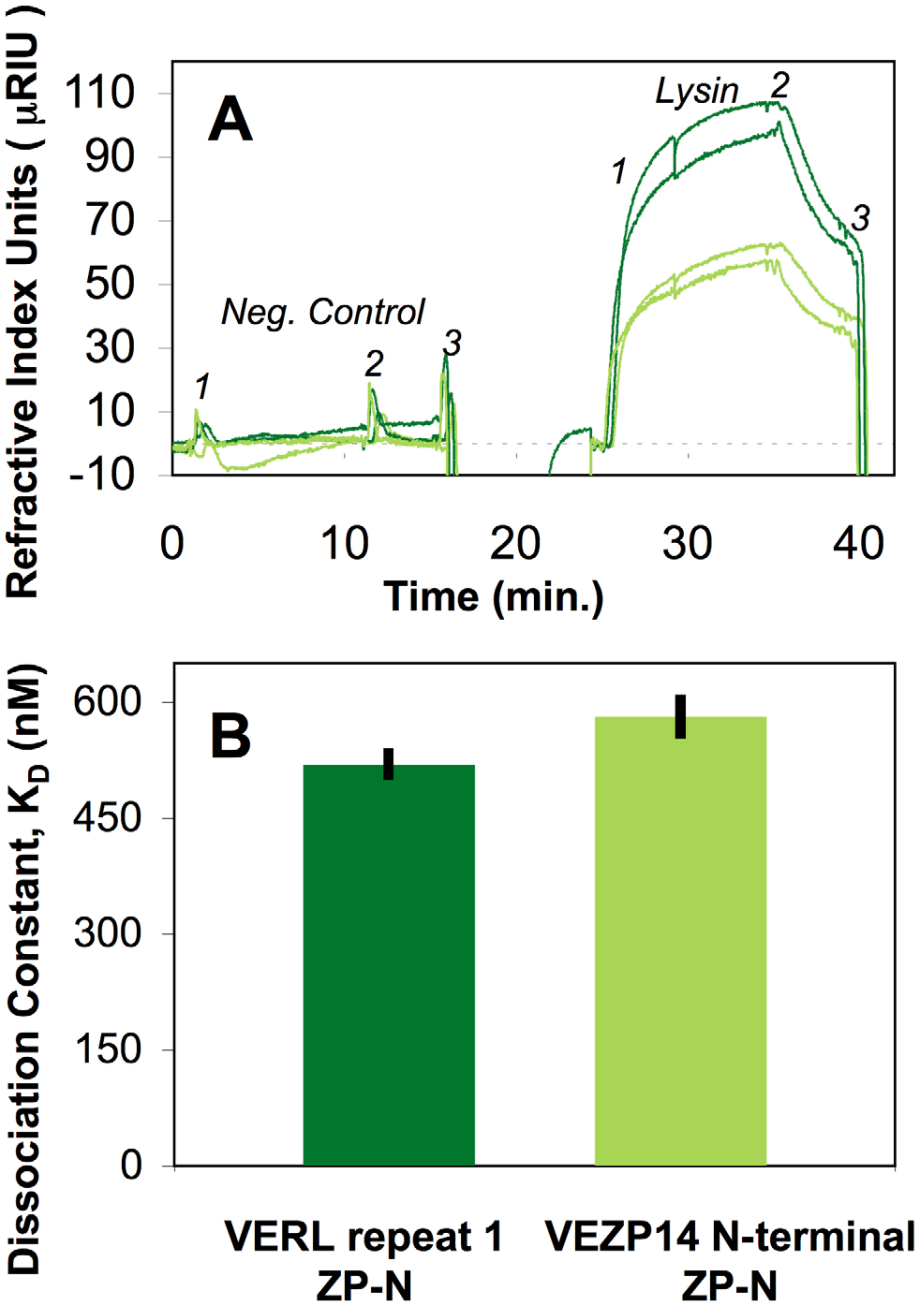
ZP-N from VERL and VEZP14 bind lysin with comparable affinity. The ZP-N motif from VEZP14 and the first repeat of green abalone VERL were cloned, expressed in *Drosophila* S2 cells (Invitrogen, Carlsbad, CA), and affinity purified (Figure S2). (A) An in-house surface plasmon resonance (SPR) detection system [54] was used to estimate binding of green abalone sperm lysin to surfaces coated with VERL (dark green) or VEZP14 (light green) as measured by refractive index (two replicates each). Numbered shoulders of the SPR curves indicate the intervals used to calculate the association (k_a_, 1–2) and dissociation (k_d_,2–3) rates via Scrubber2 (Center for Biomolecular Interaction Analysis, University of Utah). (B) Mean dissociation constants calculated from SPR data (K_D_ = k_d_/k_a_) show lysin has slightly higher, though comparable, affinity for the ZP-N motif from VERL (520 nM) and VEZP14 (580 nM; lower K_D_ values indicate higher affinity), demonstrating the ZP-N motif of egg coat proteins alone is sufficient to bind sperm lysin.

SPR shows binding between green abalone lysin and expressed ZP-N proteins from VERL and VEZP4 are qualitatively similar. Though slightly more lysin was bound by VERL (as evidenced by a consistently higher absolute refractive index over replicate channels), the kinetics of binding between lysin and both ZP-N motifs over association and dissociation intervals are comparable, yielding equilibrium dissociation constants (K_D_) of 5.2×10^−7^ and 5.8×10^−7^ M for VERL and VEZP14, respectively (Figure 2A, 2B). A negative control of similar size and charge as lysin showed no evidence of binding to ZP-N motifs, validating the specificity of the interaction. Because binding kinetics are similar (lysin-VERL k_a_ = 4.1×10^−4^ M^−1^ s^−1^, k_d_ = 2.1×10^−3^ s^−1^; lysin-VEZP14 k_a_ = 3.7×10^−4^ M^−1^ s^−1^, k_d_ = 2.1×10^−3^ s^−1^) the small quantitative differences in the absolute amount of lysin bound (Figure 2A) likely reflects differential functionality of expressed ZP-N due, e.g., to higher rates of misfolding among transformed cell lines. In sum, our binding assays show that (*i*) ZP-N motifs from VERL or VEZP14 alone are sufficient to bind abalone lysin, and (ii) the kinetics of binding between lysin and ZP-N from these VE proteins are similar, yielding affinities that differ quantitatively very little (∼10%) based on *in vitro* binding assays.

### Sites under positive selection are similar between VERL and VEZP14

**Figure 3:**
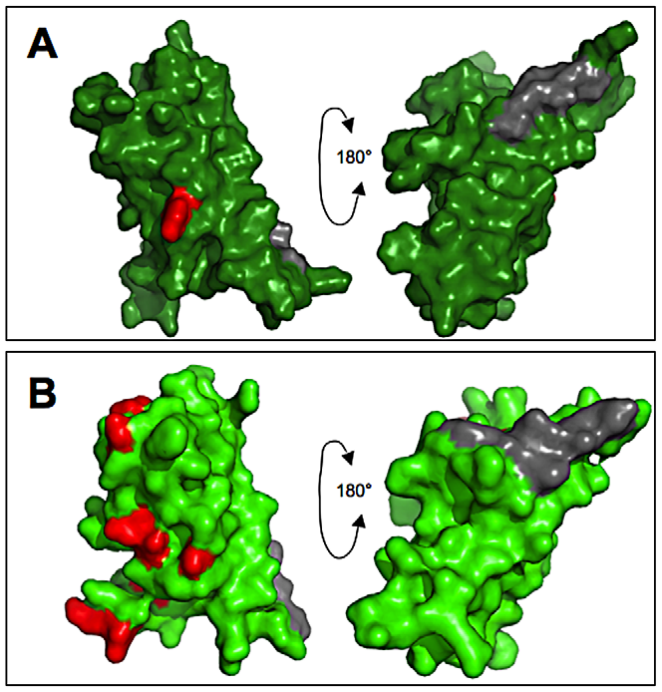
Positively selected residues occupy the exposed surface of egg coat proteins. Positively selected sites of the (A) VERL and (B) VEZP14 ZP-N motifs were inferred among 8 North Pacific abalone taxa using the Bayes Empirical Bayes (BEB) procedure of codeml [49], and sites with high posterior probabilities (>95%) were mapped to the respective structural models [22] using PyMol [52]. The positions of positively selected sites are given with reference to the complete coding sequences of VEZP14 and VERL (repeat 1 only) from red abalone ([19] and [15], respectively). For VERL, the single site under positive selection identified with high posterior probability (V42) was inferred from the first two full VERL repeats, as statistical power from concatenated ZP-N motifs alone is insufficient to allow for predicting sites under selection via BEB (Table 1). This site occupies the same position of the structural model for ZP-N from VEZP14 (S171), with the majority of the remaining seven residues predicted to be under positive selection (N173, R187, E209, I218, L220, K228, and A233) occuring on the same exposed surface opposite the E′ extension (grey fill) thought to facilitate antiparallel pairing among intermolecular ZP-N motifs [23].

The residues targeted by positive selection appear to be much the same between ZP-N motifs of VERL and VEZP14. While positive selection on VERL is somewhat weaker relative to VEZP14, as evidenced by a significant test statistic only when analyzing full repeats 1 and 2 and lower *d*_N_/*d*_S_ (ω) for sites predicted to be under positive selection (Table 1), codon substitution models identify the same proportion of positively selected sites (19%). Moreover, positively selected residues predicted with high posterior probabilities occupy the exposed face of structural models for VERL and VEZP14 ZP-N (Figure 3), corresponding to the face opposite an *E′* extension likely to mediate antiparallel pairing among ZP-N motifs [23]. Thus both the proportion and proximity of positively selected residues is consistent with SPR assays showing lysin binds both ZP-N's with high affinity.

### Does *in vitro* affinity between lysin and ZP-N reflect *in vivo* binding kinetics?

To a first approximation, the binding affinity we report between lysin and the expressed ZP-N motif of VEZP14 is likely to also be reflective of *in vivo* binding during abalone fertilization. VEZP14 contains a single N-terminal ZP-N motif isolated from the ZP polymerization domain by ∼20 tandem repeats of a hypothetically unstructured 7-residue Thr/Pro-rich region not found in VERL [19]. Yeast adhesion proteins are often similarly arranged, recognition domains extended above the cell surface by these unstructured “stalks” which in yeast facilitate unobstructed binding with ligand [see [26], [27] for reviews]. If VEZP14 is arrayed at the abalone VE surface similar to such yeast adhesion proteins, and absent inclusion in a complex of VE proteins mediated by intermolecular ZP-N pairing (see below), the kinetics of binding between lysin and the ZP-N motif of VEZP14 is likely to be fairly well represented by our SPR assay.

In contrast, *in vivo* kinetics of binding between lysin and VERL may be considerably more complex due to several factors. First, VERL is thought to form highly condensed fibers within the VE via inter-molecular pairing of VERL repeats [16], suggesting binding kinetics could be strongly influenced by steric inhibition. Second, we were only able to examine affinity between lysin and ZP-N from the first repeat of VERL, which contains an array of ∼22 tandem repeats [15] each with a ZP-N motif [22]. ZP-N from the second repeat is distinct in sequence (e.g., 39/56% amino acid sequence identity/similarity for green abalone), and ZP-N from internal homogenized repeats are further differentiated (88/94% identity/similarity with repeat 2). This is analogous to the yeast mating recognition protein α-Agglutinin, which contains three immunoglobulin (Ig) like domains, one of which (IgIII) was recently shown to have structural homology with ZP-N from VERL and VEZP14 [22]. SPR identifies both high (K_D_∼10^−9^ M) and low (K_D_∼10^−4^ M) affinity between α-Agglutinin and its ligand Aga2p [28], likely due to interactions among different subunits [27]. Relative to yeast Agglutinins, binding between lysin and egg coat ZP-N exhibits an intermediate affinity (K_D_∼10^−7^ M) that we predict could be greater for internal repeats based on VE dissolution kinetics. Binding assays using intact VERL isolated from VEs indicate positive cooperativity consistent with VE dissolution assays showing the rate limiting step is lysin's initial binding with VE proteins [10]. For VERL, lysin is thought to bind to the N-terminal repeat first [16] (corresponding the ZP-N motif we assayed via SPR) – an increase in the rate of VE dissolution following this step suggests either higher rates of association (k_a_) or lower rates of dissociation (k_d_) between lysin and internal VERL repeats, leading to a net increase in affinity (K_D_).

### Do VERL and VEZP14 have similar functions mediating lysin's dissolution of VEs?

The raised VE of abalone eggs presents a physical barrier mediating sperm access, commonly viewed as one component of the redundant polyspermy blocks which function to avoid developmental arrest of the embryo due to multiple sperm fusing with the egg nucleus [29], [30]. Ultrastructural studies show sperm lysin creates a hole in the VE for sperm entry by decondensing compact fibers [31] which were previously shown to contain VERL complexes held together by hydrophobic and electrostatic interactions [10], [16]. Because VEZP14 is (*i*) also a major component of the abalone VE [19], (*ii*) under strong positive selection consistent with lysin's species-specific VE dissolution [32], and (*iii*) can bind lysin at comparable affinity as VERL via the ZP-N receptor motif, VEZP14 is a strong candidate to also be a component of the VE's molecular mechanism mediating sperm entry. One possibility is that VEZP14 is an integral component of the compact VE fibers that decondense upon lysin binding. For example, VEZP14 dimers could form a “molecular zipper” through antiparallel pairing of ZP-N anchored to a scaffold of ∼30 other VE proteins via their ZP polymerization modules [19], much like the model for VERL fibers [16]. Alternately, VEZP14 could pair with ZP-N motifs across VERL repeats to form more complex multimers. Such structural complexity would provide an additional dimension to lysin's role in VE dissolution beyond the relatively straightforward kinetics of ligand/receptor binding, reminiscent of the view that supramolecular structure as opposed to protein sequence *per se* mediates sperm recognition of mammalian egg coat proteins [33].

### Contrasting patterns of molecular co-evolution with lysin

The contrasting patterns of molecular co-evolution between lysin and its duplicate egg coat receptors VERL and VEZP14 among North Pacific abalone presents a challenge to these and other models of abalone fertilization for which VERL and VEZP14 have functional redundancy. The positive correlation in *d*_N_/*d*_S_ between lysin and VERL's ZP-N motifs from repeats 1 and 2 (Figure 1A) is consistent with expectations of cognate sperm and egg receptors when strong directional selection is driven by sexual conflicts that develop between males and females over different fertilization optima [5], [34], and has been demonstrated previously for lysin and VERL [9]. In contrast, ZP-N from VEZP14 shows a corresponding decrease in *d*_N_/*d*_S_ as lysin's divergence accelerates (Figure 1B), essentially the mirror opposite as for VERL despite evidence from green abalone of similar binding kinetics (Figure 2A, 2B). It's possible that this negative correlation indicates VEZP14's function is entirely distinct from VERL's. For example, VEZP14 could function in host avoidance of gamete pathogens [35], explaining its rapid divergence along lineages distinct from those where lysin evolves rapidly via the dynamics of a characteristic host-pathogen co-evolutionary chase [36]. However, this seems unlikely given clear evidence that positive selection specifically targets residues occupying the same face of the ZP-N motif of VEZP14, and lysin binds ZP-N of VERL and VEZP14 with similar affinity. Alternately, the other abundant and rapidly evolving sperm acrosomal protein sp18 [37] might preferentially bind with ZP-N of VEZP14. But previous binding studies suggest sp18's affinity for both VERL and VEZP14 are very weak [19], and sp18 has no evident effect on the VE, instead localizing to the egg plasma membrane surface [37].

### Molecular mimicry predicts the co-evolution of abalone fertilization proteins

An intriguing model of VERL and VEZP14 function that could resolve their contrasting patterns of molecular co-evolution with lysin is derived by incorporating concepts of molecular mimicry with sexual conflict over fertilization optima. Mimicry is a central topic in evolutionary biology, and phenotypic mimics (of warning coloration or as camouflage) are seminal examples of natural selection [38]–[41]. Sexual mimicry, where females mimic the genitalia or secondary sexual traits of males in response to sexual antagonism, is well documented and thought to evolve in response to sexual antagonism [42], [43]. To our knowledge, sexual mimicry has never been proposed to extend to the molecular level, but the general phenomenon of molecular mimics or decoys is well established in immunology [44]. Molecular decoys function as mimics of ligand-targeted receptors, except that their downstream effector functions are entirely lost and they retain only the ability to bind ligand. Decoys thus provide a way to decrease the concentration or effectiveness of a ligand without suffering the pleiotropic effects of modifying the target receptor itself. Under this model VERL and VEZP14 might have similar interactions with lysin, but VEZP14 is not a structural component of the VE *per se* as binding with lysin affects VE integrity only indirectly by modifying the effective concentration of lysin available for binding with VERL. Importantly, paralogs such as VERL and VEZP14 [19] may be particularly good candidates from which molecular decoys evolve, as this form of mimicry is essentially a special case of sub-functionalization following gene duplication [45].

**Figure 4:**
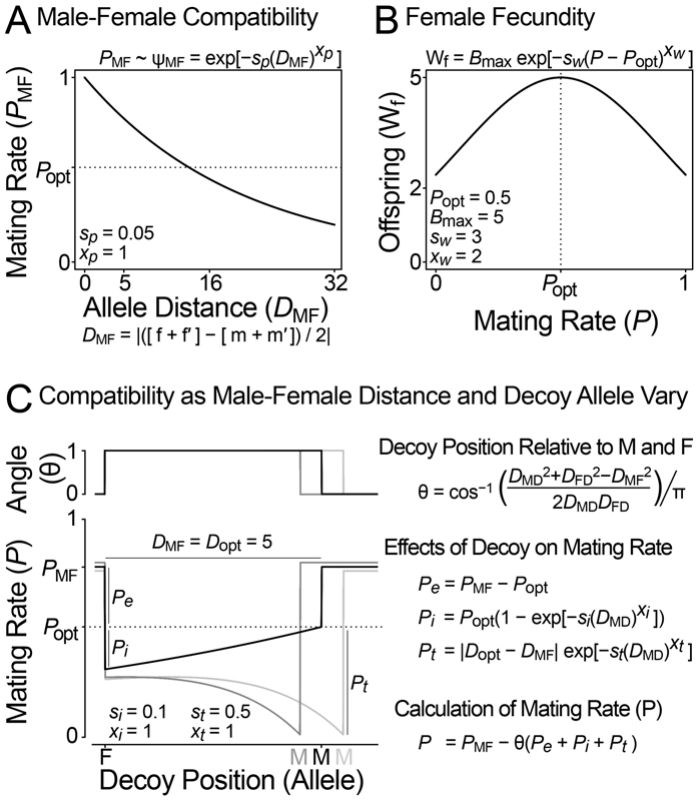
Model of sexual conflict with a female decoy. (A and B) Hayashi et al's original diploid model of sexual conflict [56]. (A) The mating rate (P) depends on the phenotypic distance between male and female reproductive loci (D_MF_), which is determined additively from the allelic values of the male and female loci (f|f′ and m|m′). (B) The number of offspring per female (W_f_) decreases from her maximum (B_max_) as her mating rate deviates from the optimal female mating rate (*P*_opt_). The scale (s_w_) and exponent (x_w_) parameters describe the shape of this relationship. Males maximize their reproductive output at P = 1, females at P = P_opt_, and thus they are in conflict over optimal mating rate. (C) A model describing the influence of a female decoy on mating rate. A female-expressed decoy locus modifies the mating rate of a male-female pair (P_MF_) by *Pe*, *Pi* and *Pt*. The decoy only influences mating rate when its additively determined phenotypic value is between those of the male and female (θ). The decoy's influence on mating rate depends in part on the male-decoy distance. *Pe* (decoy effectiveness) reduces mating rate to the female's optimal value. *Pi* (decoy interference) further reduces mating rate when the decoy and female are similar. *Pt* (the magnitude of female-decoy interference) depends on an optimal male-female distance for decoy function (*D*_MF_ = *D*_opt_). The black line shows the effect of the decoy when D_MF_ = D_opt_: as the distance between decoy and male decreases, the decoy causes mating rate to approach the female optima P_opt_. Grey lines show how the decoy reduces mating rate (*Pt*) when the male and female loci are not separated by the optimal distance for decoy function: *D*_MF_ = *D*_opt_±0.5. The scale (*si*, *sd*) and exponent (*xi*, *xd*) parameters control the shape of the *Pi* and *Pt* curves as the position of the decoy varies.

**Figure 5:**
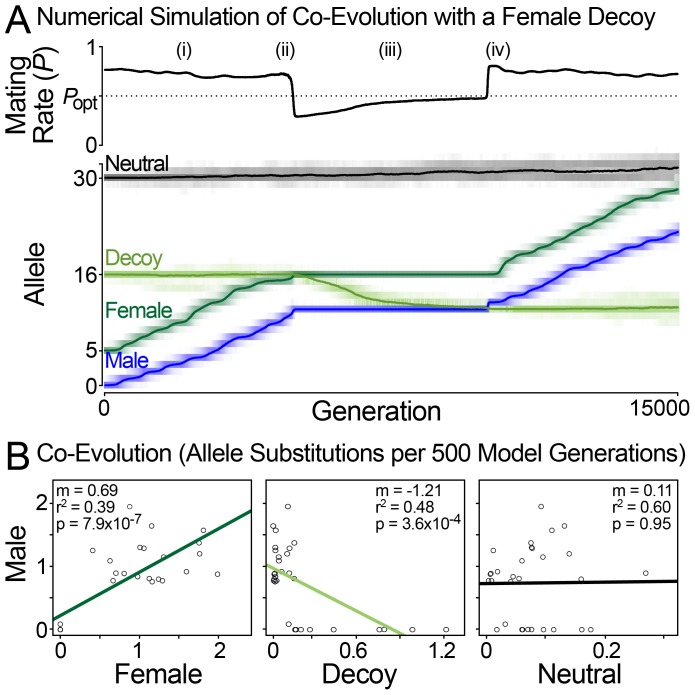
Population simulation of sexual conflict with a female decoy. (A) A representative run of the simulation showing how the decoy influences mating rate and male-female co-evolution. Simulation parameters: N = 10 000, μ = 5.0×10^−5^ per locus, each female encounters 20 males per generation. Model parameter values are shown in Figure 4. Evolutionary dynamics: (*i*) Mating rate is above *P*_opt_, and the male and female are in a co-evolutionary chase. (*ii*) The decoy becomes effective when it is between the male and female loci. The decoy causes the mating rate to drop below *P*_opt_, and male-female coevolution stops. (*iii*) The decoy evolves toward the male to reduce interference with the female, and mating rate approaches *P*_opt_. (*iv*) The decoy is no longer between the male and female loci and is thus ineffective. Mating rate increases above *P*_opt_, and male-female co-evolution resumes. *Summary*. When the decoy's phenotype is not between those of the male and female, mating rate exceeds P_opt_ and the male and female co-evolve. When the decoy is between the male and female, it causes the mating rate to drop below P_opt_. This stops antagonistic co-evolution, because in this circumstance both males and females benefit from an increased mating rate. When between the male and female, the decoy is driven toward the male locus by selection to reduce interference with the female. Thus, the decoy evolves rapidly when males and females evolve slowly and vice versa. (B) Patterns of co-evolution predicted by the model match those of natural abalone fertilization proteins. Plots of allele substitutions per 500 model generations show correlated male-female evolution and anti-correlated male-decoy evolution. Values are from the run shown in (A).

We created a model of sexual conflict that can generate the correlations observed among abalone fertilization proteins by assuming that VEZP14 acts as a molecular decoy of VERL. We model the evolutionary dynamics of three diploid genes (male, female, and decoy) in response to sexual conflict over mating rate. In the model, the male (lysin) and female (VERL) proteins co-evolve with one another because of a conflict over optimal mating rate, as expected and previously observed in simulations and natural phylogenetic comparisons [5], [34]. Successful males have high mating rates. Selection favors male alleles with values that are close to the average female allele, because in the model mating rate increases as the distance between male and female decreases (Figure 4A). Females prefer an intermediate mating rate (Figure 4B). This conflict causes co-evolution of the male and female loci. As males advance females retreat, evolving alleles that are farther from the average male value. During co-evolutionary chase, the average mating rate is generally between the male and female optima: males would prefer a higher mating rate, females lower (Figure 5A(*i*)).

We imagine that a decoy locus could modify this sexual conflict over mating rate as follows. The female and decoy loci are initially similar, either because the decoy arose as a duplicate of the existing female receptor (as seems to be the case for VEZP14 and VERL) [19], or because the female receptor and an existing non-reproductive receptor evolve to be similar (as may be the case for duplicates that are homogenized by concerted evolution). The decoy reduces mating rate toward the female's optima (in this case by binding excess lysin and reducing fertilization rate). It is probably not reasonable to expect that the decoy functions perfectly, and so we express this as interference between the female and decoy that reduces mating rate below P_opt_ (Figure 4C, Figure 5A(*ii*)). This has two effects (Figure 5A (*iii*)): *(i)* it stops male-female co-evolution since both now benefit from an increased mating rate; *(ii)* it provides an impetus for the decoy to evolve traits that optimize the male-female interaction (to tailor mating rate to P_opt_). For example, the decoy could evolve a different affinity for the male ligand (or in another context, change its pattern of regulation or spatial location). As differences between the true female receptor and the decoy accumulate, males evolve the ability to preferentially target the true receptor, causing the decoy to become ineffective (Figure 5A(*iv*)). This increases a male's mating rate at the cost of each individual female's fecundity, and co-evolution resumes as per the general model of sexual conflict. As the co-evolutionary chase proceeds, male-female phenotype space may re-encounter the decoy leading to its indefinite maintenance via recurrent positive selection.

Numerical simulations under this model always generate positive correlations between the allelic substitution rate of male and female loci (Figure 5B). The neutral locus does not show evidence of correlated evolution with any other locus (0 of 20). Anti-correlation of male and decoy loci occurs in a majority of model runs (11 of 20, Fisher's exact test P = 0.0035; Figure 5B). Thus our model mirrors at a population level the patterns observed for lysin, VERL, and VEZP14 among species of North Pacific abalone (Figure 1A, 1B). Our population simulations also identify several key conditions necessary for VEZP14 to function as a decoy of VERL during fertilization. First, a key aspect of our model is that the decoy reduce mating rate toward the female's optimum (Figure 4C). The decoy can only be effective if it does not harm the female. The *Pi* parameter (which describes female-decoy interference) must take a value that causes female fitness to be greater than at the unmodified mating rate *P*_MF_ (*Pi*<*Pe*, assuming *Pe* = *P*_MF_ − *P*_opt_). Second, we aim to model a process that could continue indefinitely, rather than approaching equilibrium. Selection for the decoy to evolve exists because *P* approaches *P*_opt_ as the decoy approaches the male (Figure 4C). To avoid all three loci collapsing to a single allelic value, we keep the male and female loci apart by assuming that there is an optimal male-female distance for decoy function (*D*_opt_). The magnitude of female-decoy interference (*Pt*) increases as the male female distance *D*_MF_ deviates from *D*_opt_. Third, we model this process in one trait dimension; the decoy is either between the male and female, or not (θ = 1 or 0, respectively) (Figure 4C). When the male can discriminate the decoy and resume co-evolution with the female, the penalty for deviation from *D*_opt_ must be large (*Pt*∼*P*_opt_, when |*D*_MF_ – *D*_opt_| = 0.5), otherwise the decoy will begin a co-evolutionary chase with the male. Even at large *Pt*, the decoy does briefly chase the male in 9 of 20 simulations (those that do not show overall evidence of anti-correlated evolution). Interestingly, among North Pacific abalone lysin and VEZP14 do sometimes take high values on the same branch (Figure 1B) despite the predominance of anti-correlation. Thus discrete instances of male-decoy co-evolution may exist even when molecular decoys function as our model predicts.

### Conclusions

We present an example of the value of evolutionary analyses in the interpretation of functional phenotypic data. On the simplest level, evolutionary analyses as straightforward as the tests of correlated *d*_N_/*d*_S_ we employ serve to focus genetic or biochemical investigation on the discrete functional domains that mediate molecular interactions. However, because these methods reflect the long-term dynamics of distinct evolutionary lineages, they may also reveal unique modes of interaction not evident in traditional functional assays. We show that the ZP-N motifs of VERL and VEZP14 bind sperm lysin with similar affinity, so the naïve interpretation is that they may also function similarly as duplicate egg coat receptors of lysin. But evidence of anti-correlated co-evolution supports a more complex interpretation: VEZP14 may function as a VERL mimic to bind excess lysin, thereby modifying the dynamics of sexual conflict over fertilization rate. This novel hypothesis, that molecular mimicry functions in addition to other well-established polyspermy blocks, promises new insights and approaches to understanding the extraordinary molecular diversity of reproductive genes and their remarkable patterns of evolution.

## Materials and Methods

### DNA sequencing

Protein coding DNA sequences for sperm and egg genes from 8 North Pacific abalone species [17] used here for evolutionary analyses were previously published, or were newly obtained from ovary transcripts. Abalone lysin (accessions M34388-9, M59968-72, M98875), and VERL repeat 1 and 2 (AF453553, AF490760-6) alignments are from [18], [46]. Publicly available VEZP14 sequences (GQ851905, GQ851925-7) were aligned by eye with sequences from 4 additional abalone taxa (*H. wallalensis*, *H. sorenseni*, *H. cracherdii*, and *H. kamtschatkana*) newly obtained from ovary transcripts of the respective taxa using an identical sequencing approach as in [19] (NCBI accession numbers JX290365-8). Alignment of all ZP-N motifs followed the structural homology model of ZP-N presented in [22], and there are no gaps in the alignment, thus there is a very low probability of comparing non-homologous codons or residues in our statistical analyses. Genes used as control loci in analyses of evolutionary rate correlations (Figure S1; cellulase: AB125892, FJ940284-90, FJ940292; haemocyanin: FJ940383-90) were described previously for this purpose [9].

### Co-evolutionary analyses

To test for correlated rates of evolution between lysin and putative binding motifs of VERL and VEZP14, we fit linear regression models to point estimates of branch-specific *d*_N_/*d*_S_ values. The general approach was developed previously from know cases of protein-protein interaction [8], [9], and serves as a conservative but robust means of testing for correlation in *d*_N_/*d*_S_ in the absence of genome-wide data necessary for statistical validation of more powerful methods [8], [47]. Point estimates of *d*_N_/*d*_S_ for lysin and ZP-N motifs from VERL and VEZP14 were made using the branch model of [48] implemented in the PAML computer package [49] following the tree topology and all other conventions exactly as in [9]. Signal peptides were excluded, and boundaries of ZP-N motifs followed [22]; for VERL, ZP-N motifs from the first two repeats that combined are the only regions with evidence of positive selection [9], [18] were concatenated for the analysis (see Table 1), but were also tested individually. Linear regressions were fit to point estimates of *d*_N_/*d*_S_ that were either unweighted, or which were weighted by the length of the respective branch as in [9], using the R statistical package [50], with correlated rates inferred as a significant slope parameter (p<0.05). Correlations were also estimated between lysin or ZP-N motifs and control loci, including the C-terminal ZP domain of VEZP14.

Mapping of positively selected residues onto structural models was used to identify potential regions of binding between lysin and ZP-N motifs of VERL or VEZP14. Sites models of codon evolution were first used to test for evidence of positive selection on ZP-N motifs, after which posterior probabilities of residues under positive selection were inferred via the Bayes Empirical Bayes algorithm [51] implemented in the PAML software package [49]. The VEZP14 alignment was identical to those for tests of correlated evolution; for VERL the entire sequence of repeats 1 and 2 were analyzed, as test statistics from ZP-N motifs alone were not statistically significant (see Table 1). Positively selected sites with posterior probabilities >95% were located on the structural models of ZP-N motifs from VERL and VEZP14 [22] using the PyMol software package [52].

### Expression of ZP-N motifs

ZP-N motifs from *Haliotis fulgens* [chosen because of extensive previous biochemical and structural studies of reproductive proteins; see, e.g. [53],] VERL and VEZP14 were expressed *in vitro* and proteins purified for binding assays with lysin. PCR primers incorporating 5′-BglII and 3′-ApaI restriction sites were designed from *H. fulgens* sequence to amplify ZP-N motifs from VERL repeats 1, 2, and 3 individually, as well as VEZP14 using standard PCR protocols. PCR products for VERL repeat 1 and VEZP14 ZP-N motifs were verified via Sanger sequencing, but repeated attempts at cloning PCR products from VERL repeats 2 and 3 were unsuccessful. PCR products were digested with BglII and ApaI following manufacturers guidelines (NEB; Beverly, MA), and cloned into vector pMT/BiP/V5His v.A before transfecting S2 cells following manufacturers guidelines for the *Drosophila* Expression System (Invitrogen; Carlsbad, CA).

ZP-N proteins were purified from transfected S2 cell culture media using immobilized metal affinity chromatography (IMAC). Serum free media (250 mL) containing transfected S2 cell cultures at a density of ∼5×10^4^ cells/ml were grown 24 hours, followed by induction of protein expression using 750 µM CuSO_4_ for an additional 24 hours. Cells were precipitated from the media and discarded, protease inhibitors (Novagen; Madison, WI) and 1 ml of chelating sepharose (GE Healthcare; Upsalla, Sweden) added to the supernatant and stirred overnight at 4°C to allow binding with a C-terminal 6X His tag. Sepharose-bound proteins were washed twice with 50 ml PBS buffer (80 mM Na_2_HPO_4_, 20 mM Na_2_H_2_PO_4_, 100 mM NaCl, pH 7.5), twice with PBS buffer supplemented to 500 mM NaCl, and bound protein eluted by sequential addition of 10 ml imidazole at increasing concentration (10, 50, and 250 mM imidazole; 100 mM Tris, pH 8.0). Select fractions were analyzed for expression products in the range of 17.1 and 16.6 kilo Daltons (kDa; VERL and VEZP14, respectively) via SDS PAGE as in [10], and by probing Western blots with mouse monoclonal antibodies to the C-terminal V5 epitope per manufacture's guidelines (Invitrogen; Carlsbad, CA). Fractions with evidence of expression proteins in the target size range were pooled, dialized overnight at 4°C into two 500 ml changes of PBS buffer, and protein concentrations measured via BCA (Thermo Scientific; Rockford, IL). Purified proteins were confirmed as VERL repeat 1 or VEZP14 ZP-N motifs based on matching of peptide mass spectra using shotgun proteomics as in [19], but analyzing 1 µg of trypsin-digested protein via reversed-phase HPLC in a 1 hour water:acetonitrile gradient. Differential glycosylation was tested as an explanation for laddered VERL ZP-N bands (see Figure S2) by enzymatic de-glycosylation of 25 µg of protein using PNGaseF (Sigma; St. Louis, MO) and assessing mobility shifts via SDS PAGE.

### Binding assays

Quantitative measurements of binding affinity between lysin and ZP-N motifs from VERL and VEZP14 were made using an in-house surface plasmon resonance (SPR) detection system described previously [54]. Gold sensor surfaces were coated overnight at 4°C with 2 µg IMAC purified VERL repeat 1 or VEZP14 ZP-N (two replicates each), and rinsed with PBS buffer prior to SPR analysis. Injected analytes included green abalone lysin as well as chicken lysozome (Sigma; St. Louis, MO), a negative control of similar size and charge as abalone lysin. Lysin was purified from green abalone testis using ion exchange chromatography exactly as in [55], dialized overnight at 4°C into two changes of HEPES buffered seawater (HBSW; filtered seawater, 10 mM HEPES, pH 7.8), and diluted to a concentration of 40 µg/ml with HBSW supplemented with 0.5% Triton X-100 and 1 mg/ml bovine serum albumin (Sigma; St. Louis, MO). The sample loop of the SPR system was equilibrated with supplemented HBSW at 25°C, followed by injection of 1 ml of negative control or lysin analytes flowed over sensors at 70 µl/min for 10 minutes (association interval), after which analyte was removed and the sample loop flushed with supplemented HBSW for 5 minutes (dissociation interval). ZP-N coated sensor surfaces were regenerated between injections by removing bound analyte with 100 mM glycine (pH 2.8) and equilibrating with supplemented HBSW for 10 minutes. Measurements of refractive index units (RIU) were then used to calculate the association (k_a_) and dissociation (k_d_) rates from association and dissociation intervals of SPR data by curve fitting using non-linear least-squares regression, implemented via Scrubber2 (Center for Biomolecular Interaction Analysis, University of Utah).

### Model of sexual conflict with a female decoy

We extended the diploid two-locus sexual conflict models of [56] by incorporating a female decoy, a locus that modifies mating rates toward the female's optima. The alleles of the male, female, and decoy genes are represented by numbers between 1 and 50, and the distances between these numbers determine the mating rate of a given male and female pair. We assume that alleles determine the phenotype additively for all loci, since this is most likely to result in continuous male-female co-evolution [56]. Figure 4A and 4B describe how a difference in optimal mating rate can result in sexual conflict. The mating rate of a male-female pair depends on the phenotypic distance (*D*_MF_) between the male and female as determined additively by alleles of sex-limited male (m/m′) and female (f/f′) reproductive loci. Small phenotypic distance values cause high mating rates. The number of offspring per female decreases as her mating rate (*P*) deviates above or below the optimal female mating rate (*P*_opt_). The optimal male mating rate is 1, and males increase mating rate by evolving alleles that are similar to females. Above *P*_opt_ females are selected to reduce mating rate by evolving away from males and a co-evolutionary chase ensues [56]. Below P_opt_ both females and males benefit from an increased mating rate. Figure 4C shows how a decoy locus can modify the mating rate in line with the female's optima. The decoy is only active when its phenotypic value lies between the male and female (θ = 1). The decoy's effect on mating rate is broken down into three components (*Pe*, *Pi*, and *Pt*). *Pe* (decoy effectiveness) reduces mating rate to *P*_opt_. *Pi* (female-decoy interference) further reduces mating rate when the decoy is similar to the female. Interference decreases as the decoy approaches the male, and the mating rate thus approaches *P*_opt_. *Pt* (the magnitude of female-decoy interference) depends on the male-female distance. *D*_opt_ is the optimal male-female distance for decoy function; when *D*_MF_ deviates from *D*_opt_, the decoy interferes with the female and reduces mating rate (*Pt*). Equations and parameter values describing these relationships are in Figure 4.

### Population simulations

Simulations are run on populations of N = 10,000 individuals for 15,000 generations (Figure 5). Females encounter n = 20 males per generation and mate with a proportion of them depending on the genetic distance between male and female, and on the activity of the decoy. A neutral locus lacking any effect on mating rate is also simulated, as a control to monitor for stochastic correlations. Each locus has 50 possible alleles with initial values Male: 0, Female: 5, Decoy: 16, Neutral: 30, and a mutation rate μ = 5.0×10^−5^ per locus per generation. Other simulation parameters and equations are shown in the graph detailing the model (Figure 4). As in [56], 20 simulation runs were used to assess model behavior. Scripts for running simulations were written in Perl, and are provided in Supplement S1. For comparison with actual abalone sperm and egg proteins (lysin, VERL, and VEZP14), the change in the mean allelic value over each 500 generations of the simulation was used to estimate the rate of evolutionary change (a proxy for branch-specific *d*_N_/*d*_S_ estimates along individual phylogenetic lineages). Linear regressions were fit to point estimates as was done with the lysin and ZP-N data, with correlated rates inferred as a significant slope parameter (p>0.05).

## Supporting Information

Figure S1 Correlated rates of evolution are not a general feature among abalone genes. As in Figure 1, ratios of non-synonymous to synonymous nucleotide substitutions (*d*_N_/*d*_S_ or ω) for reproductive proteins (lysin, VEZP14) or non-reproductive (cellulase, haemocyanin) genes were estimated using the branch model of codeml [49] with gene trees following the topology of [9]. Values of ω for each branch of the VEZP14 ZP-N motif (A, B) or C-terminus ZP domain (C, D) tree are plotted on the y-axis relative to x-axis values for the corresponding branch values for (A) cellulase, (B) haemocyanin, (C) lysin, or (D) the VEZP14 ZP-N motif. Simple linear regression models either weighted (solid lines) or unweighted (dashed lines) by branch length as in [9] were fit to the data [50]. No significant correlation in *d*_N_/*d*_S_ values are seen, even when comparing among functionally distinct regions of the same gene (VEZP14, D), and the correlation in rates observed between lysin and the target ZP-N motif (Figure 1) is notably absent from the C-terminus ZP domain of VEZP14.
(TIF)

Figure S2 Affinity purification of *Drosophila* S2 expressed ZP-N motifs. The ZP-N motif from VEZP14 the first repeat of green abalone VERL were cloned, expressed in *Drosophila* S2 cells (Invitrogen, Carlsbad, CA), and successively eluted from immobilized metal affinity columns (IMAC) using three concentrations of imidazole (10, 50, and 250 mM). (A) IMAC elutions employing 50–250 mM imidazole contain a large quantity of 20–24 kDa protein (dark green box); deglycosylation using PNGaseF (Sigma, St. Louis, MO) resulted in a single protein band of approx. 17 kDa (expected size of VERL repeat 1 ZP-N is 17.1 kDa). (B) IMAC elutions employing 250 mM imidazole contain a single band of approx. 22 kDa (light green box; expected size of VEZP14 ZP-N is 16.6 kDa). Western blots confirmed the identified bands as expressed proteins from the presence of a V5 epitope tag, and shotgun proteomic analyses match mass spectra from multiple peptides of both to the respective ZP-N motifs from VERL or VEZP14. Retarded migration and results from PNGaseF experiments are consistent with N-linked glycosylation of the expressed ZP-N proteins used in binding assays (Figure 3).
(TIF)

Supplement S1 Perl scripts for running population simulations of sexual conflict with a female decoy. The files are: *decoy.pl*, the main perl script; *decoy.ctl*, the control file where simulation and model parameters can be set; and sub_*.pm, various subroutines called by the main perl script. For additional detail please contact the authors.
(ZIP)

## References

[1] Clark AG, Glanowski S, Nielsen R, Thomas PD, Kejariwal A, et al (2003) Inferring nonneutral evolution from human-chimp-mouse orthologous gene trios. Science 302: 1960–1963. 14671302 10.1126/science.1088821

[2] Nielsen R, Bustamante C, Clark AG, Glanowski S, Sackton TB, et al (2005) A scan for positively selected genes in the genomes of humans and chimpanzees. PLoS Biol 3: e170 doi:10.1371/journal.pbio.0030170. 15869325 10.1371/journal.pbio.0030170 PMC1088278

[3] George RD, McVicker G, Diederich R, Ng SB, MacKenzie AP, et al (2011) Trans genomic capture and sequencing of primate exomes reveals new targets of positive selection. Genome Res 21: 1686–1694. 21795384 10.1101/gr.121327.111 PMC3202285

[4] Evolution of genes and genomes on the Drosophila phylogeny. Nature 450: 203–218. 17994087 10.1038/nature06341

[5] Swanson WJ, Vacquier VD (2002) Rapid evolution of reproductive proteins. Nature Reviews Genetics 3: 137–144. 10.1038/nrg733 11836507

[6] Clark NL, Aagaard JE, Swanson WJ (2006) Evolution of reproductive proteins from animals and plants. Reproduction 131: 11–22. 16388004 10.1530/rep.1.00357

[7] Lyon JD, Vacquier VD (1999) Interspecies chimeric sperm lysins identify regions mediating species-specific recognition of the abalone egg vitelline envelope. Developmental Biology 214: 151–159. 10491264 10.1006/dbio.1999.9411

[8] Clark NL, Aquadro CF (2010) A novel method to detect proteins evolving at correlated rates: identifying new functional relationships between coevolving proteins. Mol Biol Evol 27: 1152–1161. 20044587 10.1093/molbev/msp324 PMC2877527

[9] Clark NL, Gasper J, Sekino M, Springer SA, Aquadro CF, et al (2009) Coevolution of interacting fertilization proteins. PLoS Genet 5: e1000570 doi:10.1371/journal.pgen.1000570. 19629160 10.1371/journal.pgen.1000570 PMC2704960

[10] Swanson WJ, Vacquier VD (1997) The abalone egg vitelline envelope receptor for sperm lysin is a giant multivalent molecule. Proceedings of the National Academy of Sciences of the United States of America 94: 6724–6729. 9192632 10.1073/pnas.94.13.6724 PMC21225

[11] Kamei N, Glabe CG (2003) The species-specific egg receptor for sea urchin sperm adhesion is EBR1,a novel ADAMTS protein. Genes Dev 17: 2502–2507. 14561772 10.1101/gad.1133003 PMC218143

[12] Harada Y, Takagaki Y, Sunagawa M, Saito T, Yamada L, et al (2008) Mechanism of self-sterility in a hermaphroditic chordate. Science 320: 548–550. 18356489 10.1126/science.1152488

[13] Lewis CA, Talbot CF, Vacquier VD (1982) A protein from abalone sperm dissolves the egg vitelline layer by a nonenzymatic mechanism. Developmental Biology 92: 227–239. 7106382 10.1016/0012-1606(82)90167-1

[14] Leighton DL (2000) The Biology and Culture of the California Abalones. Pittsburgh, PA: Dorrance Publishing Co. 216 p.

[15] Galindo BE, Moy GW, Swanson WJ, Vacquier VD (2002) Full Length Sequence of VERL, the egg vitelline envelope receptor for abalone sperm lysin. Gene 288: 111–117. 12034500 10.1016/s0378-1119(02)00459-6

[16] Kresge N, Vacquier VD, Stout CD (2001) Abalone lysin: the dissolving and evolving sperm protein. Bioessays 23: 95–103. 11135314 10.1002/1521-1878(200101)23:1<95::AID-BIES1012>3.0.CO;2-C

[17] Lee Y-H, Vacquier VD (1995) Evolution and systematics in Haliotidae (Mollusca: Gastropoda): inferences from DNA sequences of sperm lysin. Marine Biology 124: 267–268.

[18] Galindo BE, Vacquier VD, Swanson WJ (2003) Positive selection in the egg receptor for abalone sperm lysin. Proc Natl Acad Sci U S A 100: 4639–4643. 12676995 10.1073/pnas.0830022100 PMC153608

[19] Aagaard JE, Vacquier VD, MacCoss MJ, Swanson WJ (2010) ZP domain proteins in the abalone egg coat include a paralog of VERL under positive selection that binds lysin and 18-kDa sperm proteins. Mol Biol Evol 27: 193–203. 19767347 10.1093/molbev/msp221 PMC2877556

[20] Aagaard JE, Yi X, MacCoss MJ, Swanson WJ (2006) Rapidly evolving zona pellucida domain proteins are a major component of the vitelline envelope of abalone eggs. Proc Natl Acad Sci U S A 103: 17302–17307. 17085584 10.1073/pnas.0603125103 PMC1859926

[21] Jovine L, Darie CC, Litscher ES, Wassarman PM (2005) Zona pellucida domain proteins. Annual Review of Biochemistry 74: 83–114. 10.1146/annurev.biochem.74.082803.133039 15952882

[22] Swanson WJ, Aagaard JE, Vacquier VD, Monne M, Sadat Al Hosseini H, et al (2011) The molecular basis of sex: linking yeast to human. Mol Biol Evol 28: 1963–1966. 21282709 10.1093/molbev/msr026 PMC3167683

[23] Monne M, Han L, Schwend T, Burendahl S, Jovine L (2008) Crystal structure of the ZP-N domain of ZP3 reveals the core fold of animal egg coats. Nature 456: 653–657. 19052627 10.1038/nature07599

[24] Chen MH, Shen ZM, Bobin S, Kahn PC, Lipke PN (1995) Structure of Saccharomyces cerevisiae alpha-agglutinin. Evidence for a yeast cell wall protein with multiple immunoglobulin-like domains with atypical disulfides. J Biol Chem 270: 26168–26177. 7592821 10.1074/jbc.270.44.26168

[25] Vacquier VD, Swanson WJ, Lee YH (1997) Positive Darwinian selection on two homologous fertilization proteins: what is the selective pressure driving their divergence? Journal of Molecular Evolution 44: S15–S22. 9071007 10.1007/pl00000049

[26] Lipke PN, Kurjan J (1992) Sexual agglutination in budding yeasts: structure, function, and regulation of adhesion glycoproteins. Microbiol Rev 56: 180–194. 1579109 10.1128/mr.56.1.180-194.1992 PMC372860

[27] Dranginis AM, Rauceo JM, Coronado JE, Lipke PN (2007) A biochemical guide to yeast adhesins: glycoproteins for social and antisocial occasions. Microbiol Mol Biol Rev 71: 282–294. 17554046 10.1128/MMBR.00037-06 PMC1899881

[28] Venter JC, Adams MD, Myers EW, Li PW, Mural RJ, et al (2001) The sequence of the human genome. Science 291: 1304–1351. 11181995 10.1126/science.1058040

[29] Wong JL, Wessel GM (2006) Defending the zygote: search for the ancestral animal block to polyspermy. Curr Top Dev Biol 72: 1–151. 16564333 10.1016/S0070-2153(05)72001-9

[30] Gould MC, Stephano JL (2003) Polyspermy prevention in marine invertebrates. Microsc Res Tech 61: 379–388. 12811743 10.1002/jemt.10351

[31] Mozingo NM, Vacquier VD, Chandler DE (1995) Structural features of the abalone egg extracellular matrix and its role in gamete interaction during fertilization. Molecular Reproduction and Development 41: 493–502. 7576617 10.1002/mrd.1080410412

[32] Lee YH, Vacquier VD (1992) The divergence of species-specific abalone sperm lysins is promoted by positive Darwinian selection. Biological Bulletin (Woods Hole) 182: 97–104. 10.2307/1542183 29304708

[33] Dean J (2004) Reassessing the molecular biology of sperm-egg recognition with mouse genetics. Bioessays 26: 29–38. 14696038 10.1002/bies.10412

[34] Palumbi SR (2009) Speciation and the evolution of gamete recognition genes: pattern and process. Heredity 102: 66–76. 19018273 10.1038/hdy.2008.104

[35] Vacquier VD (1998) Evolution of gamete recognition proteins. Science 281: 1995–1998. 9748153 10.1126/science.281.5385.1995

[36] Woolhouse ME, Webster JP, Domingo E, Charlesworth B, Levin BR (2002) Biological and biomedical implications of the co-evolution of pathogens and their hosts. Nat Genet 32: 569–577. 12457190 10.1038/ng1202-569

[37] Swanson WJ, Vacquier VD (1995) Extraordinary divergence and positive Darwinian selection in a fusagenic protein coating the acrosomal process of abalone spermatozoa. Proceedings of the National Academy of Sciences of the United States of America 92: 4957–4961. 7761431 10.1073/pnas.92.11.4957 PMC41826

[38] Wallace AR (1889) Darwinism. London: MacMillan and Co. 494 p.

[39] Darwin C (1859) On the origin of species by means of natural selection, or, The preservation of favoured races in the struggle for life. London: J. Murray. PMC5184128 30164232

[40] Sirot LK, Brockmann HJ (2001) Costs of sexual interactions to females in Rambur's forktail damselfly, Ischnura ramburi (Zygoptera: Coenagrionidae). Animal Behaviour 61: 415–424.

[41] Saetre G-P, Slagsvold T (1996) The significance of female mimicry in male contests. American Naturalist 147: 981–995.

[42] Muller MN, Wrangham R (2002) Sexual mimicry in hyenas. Q Rev Biol 77: 3–16. 11963460 10.1086/339199

[43] Robertson HM (1985) Female dimorphism and mating behavior in a damselfly, Ischnura ramburi: females mimicking males. Animal Behaviour 33: 805–809.

[44] Mantovani A, Locati M, Vecchi A, Sozzani S, Allavena P (2001) Decoy receptors: a strategy to regulate inflammatory cytokines and chemokines. Trends Immunol 22: 328–336. 11377293 10.1016/s1471-4906(01)01941-x

[45] Force A, Lynch M, Pickett FB, Amores A, Yan YL, et al (1999) Preservation of duplicate genes by complementary, degenerative mutations. Genetics 151: 1531–1545. 10101175 10.1093/genetics/151.4.1531 PMC1460548

[46] Yang Z, Swanson WJ, Vacquier VD (2000) Maximum-likelihood analysis of molecular adaptation in abalone sperm lysin reveals variable selective pressures among lineages and sites. Molecular Biology and Evolution 17: 1446–1455. 11018152 10.1093/oxfordjournals.molbev.a026245

[47] Clark NL, Alani E, Aquadro CF (2012) Evolutionary rate covariation reveals shared functionality and co-expression of genes. Genome Research 10.1101/gr.132647.111 PMC3317153 22287101

[48] Yang Z, Nielsen R (1998) Synonymous and nonsynonymous rate variation in nuclear genes of mammals. Journal of Molecular Evolution 46: 409–418. 9541535 10.1007/pl00006320

[49] Yang Z (2000) Phylogenetic Analysis by Maximum Likelihood (PAML). 3.1 ed. London: University College London.

[50] R Development Core Team (2011) R: A Language and Environment for Statistical Computing. Vienna, Austria: R Foundation for Statistical Computinhg.

[51] Yang Z, Wong WS, Nielsen R (2005) Bayes empirical bayes inference of amino acid sites under positive selection. Mol Biol Evol 22: 1107–1118. 15689528 10.1093/molbev/msi097

[52] Schrodinger LLC (2010) The PyMOL Molecular Graphics System, Version 1.3r1.

[53] Kresge N, Vacquier VD, Stout CD (2000) The high resolution crystal structure of green abalone sperm lysin: implications for species-specific binding of the egg receptor. Journal of Molecular Biology 296: 1225–1234. 10698629 10.1006/jmbi.2000.3533

[54] Chinowsky TM, Soelberg SD, Baker P, Swanson NR, Kauffman P, et al (2007) Portable 24-analyte surface plasmon resonance instruments for rapid, versatile biodetection. Biosens Bioelectron 22: 2268–2275. 17223032 10.1016/j.bios.2006.11.026

[55] Vacquier VD, Lee YH (1993) Abalone sperm lysin: unusual mode of evolution of a gamete recognition protein. Zygote 1: 181–196. 8081815 10.1017/s0967199400001465

[56] Hayashi TI, Vose M, Gavrilets S (2007) Genetic differentiation by sexual conflict. Evolution 61: 516–529. 17348917 10.1111/j.1558-5646.2007.00059.x

