# Correction: Duplicate Abalone Egg Coat Proteins Bind Sperm Lysin Similarly, but Evolve Oppositely, Consistent with Molecular Mimicry at Fertilization

**DOI:** 10.1371/annotation/30da0688-60ec-4fc3-beb0-72a343a7a9be

**Published:** 2013-02-27

**Authors:** Jan E. Aagaard, Stevan A. Springer, Scott D. Soelberg, Willie J. Swanson

The affiliation for the fourth author was incorrect. Willie J. Swanson is not affiliated with the Department of Cellular and Molecular Medicine, University of California San Diego, La Jolla, California, United States of America, but is affiliated with the Department of Genome Sciences, University of Washington, Seattle, Washington, United States of America.

