# Supplementary figures and images for: Duplicate Abalone Egg Coat Proteins Bind Sperm Lysin Similarly, but Evolve Oppositely, Consistent with Molecular Mimicry at Fertilization
Source: PLoS Genet. 2013 Feb 7;9(2):e1003287. doi: 10.1371/journal.pgen.1003287 (PMC3567151; doi:10.1371/journal.pgen.1003287)

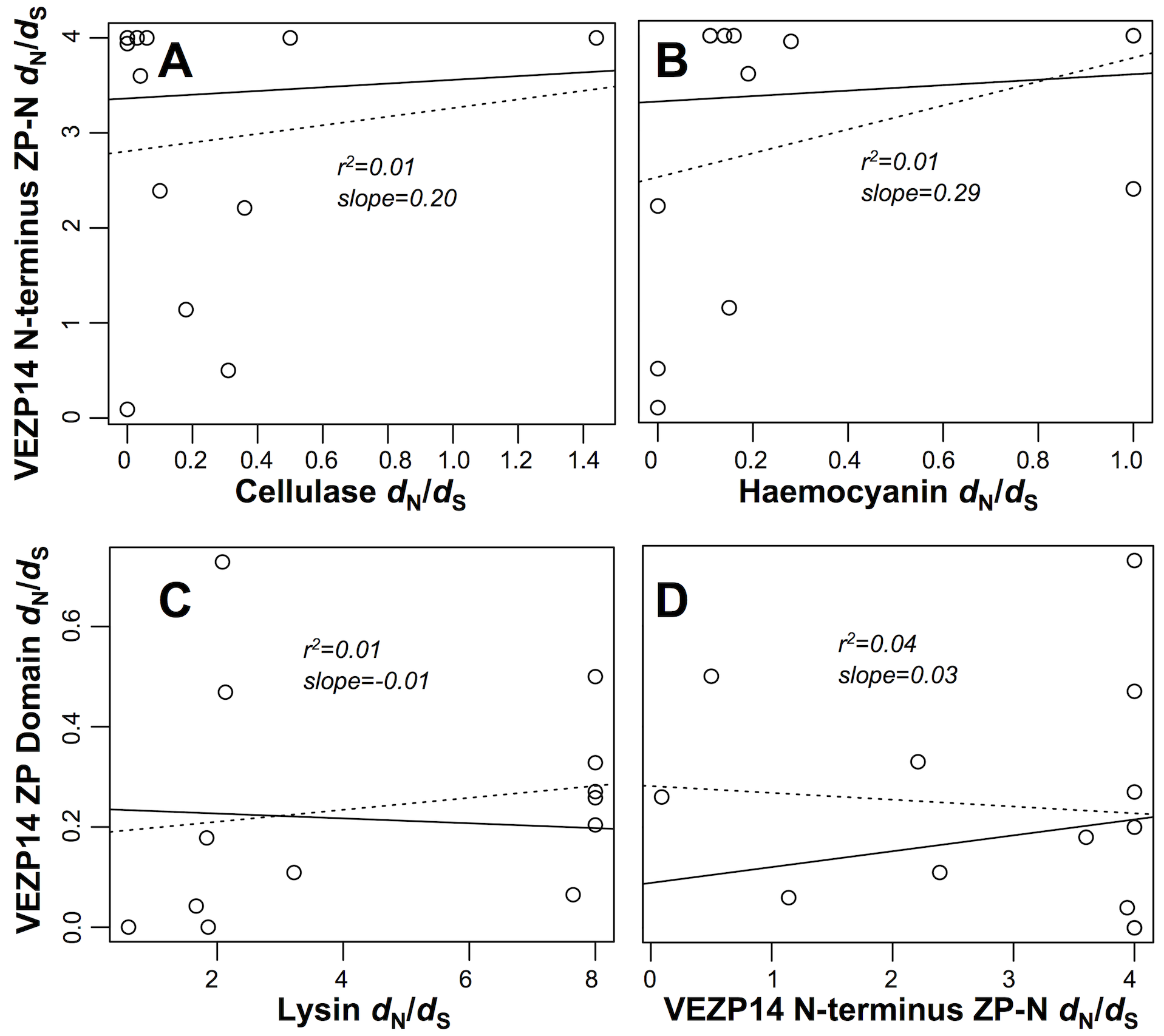

Supplement: Figure S1 — Correlated rates of evolution are not a general feature among abalone genes. As in Figure 1, ratios of non-synonymous to synonymous nucleotide substitutions (d N/d S or ω) for reproductive proteins (lysin, VEZP14) or non-reproductive (cellulase, haemocyanin) genes were estimated using the branch model of codeml [49] with gene trees following the topology of [9]. Values of ω for each branch of the VEZP14 ZP-N motif (A, B) or C-terminus ZP domain (C, D) tree are plotted on the y-axis relative to x-axis values for the corresponding branch values for (A) cellulase, (B) haemocyanin, (C) lysin, or (D) the VEZP14 ZP-N motif. Simple linear regression models either weighted (solid lines) or unweighted (dashed lines) by branch length as in [9] were fit to the data [50]. No significant correlation in d N/d S values are seen, even when comparing among functionally distinct regions of the same gene (VEZP14, D), and the correlation in rates observed between lysin and the target ZP-N motif (Figure 1) is notably absent from the C-terminus ZP domain of VEZP14. (TIF) [file pgen.1003287.s001.tif]

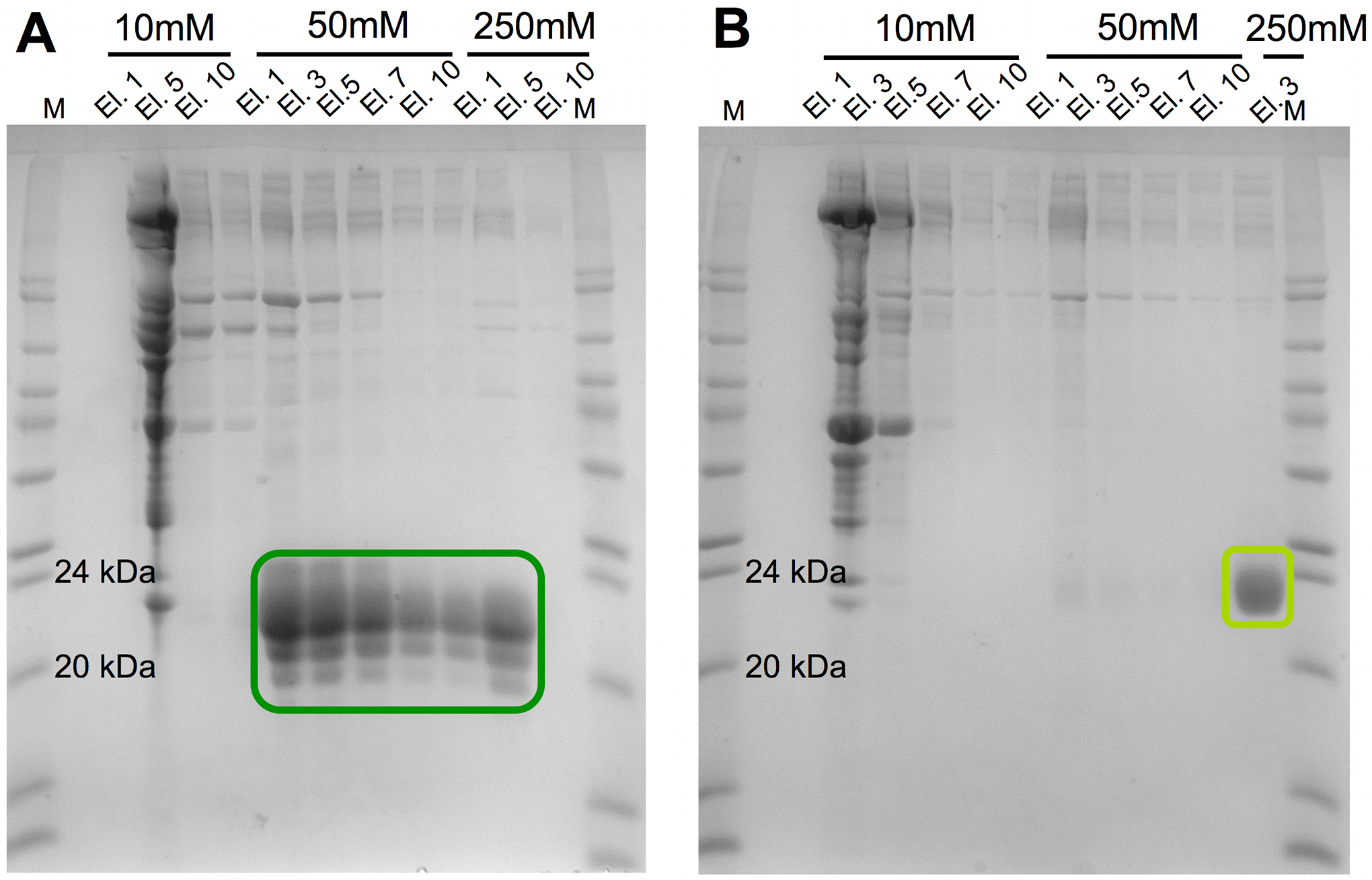

Supplement: Figure S2 — Affinity purification of Drosophila S2 expressed ZP-N motifs. The ZP-N motif from VEZP14 the first repeat of green abalone VERL were cloned, expressed in Drosophila S2 cells (Invitrogen, Carlsbad, CA), and successively eluted from immobilized metal affinity columns (IMAC) using three concentrations of imidazole (10, 50, and 250 mM). (A) IMAC elutions employing 50–250 mM imidazole contain a large quantity of 20–24 kDa protein (dark green box); deglycosylation using PNGaseF (Sigma, St. Louis, MO) resulted in a single protein band of approx. 17 kDa (expected size of VERL repeat 1 ZP-N is 17.1 kDa). (B) IMAC elutions employing 250 mM imidazole contain a single band of approx. 22 kDa (light green box; expected size of VEZP14 ZP-N is 16.6 kDa). Western blots confirmed the identified bands as expressed proteins from the presence of a V5 epitope tag, and shotgun proteomic analyses match mass spectra from multiple peptides of both to the respective ZP-N motifs from VERL or VEZP14. Retarded migration and results from PNGaseF experiments are consistent with N-linked glycosylation of the expressed ZP-N proteins used in binding assays (Figure 3). (TIF) [file pgen.1003287.s002.tif]
